# Lipopolysaccharide Transport System Links Physiological Roles of σ^E^ and ArcA in the Cell Envelope Biogenesis in Shewanella oneidensis

**DOI:** 10.1128/spectrum.00690-21

**Published:** 2021-08-18

**Authors:** Peilu Xie, Huihui Liang, Jiahao Wang, Yujia Huang, Haichun Gao

**Affiliations:** a Institute of Microbiology and College of Life Sciences, Zhejiang University, Hangzhou, Zhejiang, China; University of Minnesota

**Keywords:** lipopolysaccharide transport system, Arc regulatory system, σ^E^, cell envelope, envelope stress response, regulation

## Abstract

The bacterial cell envelope is not only a protective structure that surrounds the cytoplasm but also the place where a myriad of biological processes take place. This multilayered complex is particularly important for electroactive bacteria such as Shewanella oneidensis, as it generally hosts branched electron transport chains and numerous reductases for extracellular respiration. However, little is known about how the integrity of the cell envelope is established and maintained in these bacteria. By tracing the synthetic lethal effect of Arc two-component system and σ^E^ in S. oneidensis, in this study, we identified the lipopolysaccharide transport (Lpt) system as the determining factor. Both Arc and σ^E^, by regulating transcription of *lptFG* and *lptD*, respectively, are required for the Lpt system to function properly. The ArcA loss results in an LptFG shortage that triggers activation of σ^E^ and leads to LptD overproduction. LptFG and LptD at abnormal levels cause a defect in the lipopolysaccharide (LPS) transport, leading to cell death unless σ^E^-dependent envelope stress response is in place. Overall, our report reveals for the first time that Arc works together with σ^E^ to maintain the integrity of the S. oneidensis cell envelope by participating in the regulation of the LPS transport system.

**IMPORTANCE** Arc is a well-characterized global regulatory system that modulates cellular respiration by responding to changes in the redox status in bacterial cells. In addition to regulating expression of respiratory enzymes, Shewanella oneidensis Arc also plays a critical role in cell envelope integrity. The absence of Arc and master envelope stress response (ESR) regulator σ^E^ causes a synthetic lethal phenotype. Our research shows that the Arc loss downregulates *lptFG* expression, leading to cell envelope defects that require σ^E^-mediated ESR for viability. The complex mechanisms revealed here underscore the importance of the interplay between global regulators in bacterial adaption to their natural inhabits.

## INTRODUCTION

Bacteria live in unpredictable and harsh environments. As the outmost shell of the bacterial cell, the cell envelope constitutes the first line of defense against external assaults (1). The envelope of Gram-negative bacteria is composed of three principal layers: the outer membrane (OM), the peptidoglycan layer, and the cytoplasmic or inner membrane (IM) (2). The OM, an asymmetric phospholipid bilayer whose outside leaflet is composed of lipopolysaccharide (LPS) molecules, functions as a robust and outmost permeability barrier (1). Given its essentiality for cell viability, the biogenesis of the OM is a precisely regulated process, and perturbations and environmental threats affecting the integrity of their envelope are closely monitored (3). Under stressful conditions, envelope stress response (ESR) is activated to prevent unmanageable damages (2). Alternative sigma factor σ^E^ (RpoE), which is inactive under normal conditions because it is sequestered to the IM by its anti-sigma factor RseA, plays a primary role in mediating envelope biogenesis and ESR (4, 5). The σ^E^ signaling detects periplasmic stresses, including misfolded OM proteins and off-pathway lipopolysaccharides (LPS) input, and initiates a proteolytic cascade that results in the sequential degradation of RseA, amounting to the release of σ^E^ from the IM (6, 7). Freed cytoplasmic σ^E^ transcribes its regulon involved in synthesis, assembly, and homeostasis of OM proteins and LPS (3). In addition to σ^E^, multiple phosphorelay systems have also been discovered to mediate ESR, including two-component systems (TCSs) CpxAR and BaeRS and complex signaling transduction system Rcs (3, 8).

*Shewanella*, a group of the gammaproteobacteria that inhabit the oxic-anoxic transition zones of water columns and aquatic sediments, are renowned for their respiratory versality (9, 10). These bacteria are able to utilize a variety of compounds as terminal electron acceptors (EAs), including oxygen, fumarate, trimethylamine *N*-oxide (TMAO), dimethyl sulfoxide (DMSO), nitrate, nitrite, sulfite/thiosulfate, and metals such as iron and manganese (11, 12). Reduction of all EAs is carried out outside the cytoplasm, dependent on various reduction systems, including terminal reductases and the electron transfer chains that supply electrons (13). Given that most, if not all, of the components of the reduction systems are located in the cell envelope, establishing and maintaining the integrity of the envelope is thus vital for these biological processes. Conceivably, the σ^E^ signaling pathway dictates maintaining the cell envelope integrity and mediating ESR in Shewanella oneidensis (14). While roles of CpxAR, BaeRS, and Rcs in mediating envelope integrity and ESR of S. oneidensis remain unexplored, the anoxic redox control (Arc, ArcBA) system is unambiguously involved.

Arc, best studied in Escherichia coli, is a conventional two-component system (TCS) mediating the metabolic shift from anaerobic to aerobic conditions (15, 16). The DNA-binding response regulator ArcA regulates the expression of genes in response to oxygen availability sensed by the sensor kinase ArcB in an indirect manner, mainly by repressing genes involved in aerobic respiration (17). The S. oneidensis Arc system is atypical, as there exists a connector (HptA), a protein similar to the histidine phosphotransfer domain (HPt) of ArcB in E. coli that establishes the regulatory link between the otherwise independent sensor kinase (ArcS) and ArcA (18–20). Moreover, overlaps in the ArcA regulons of these two species are surprisingly rare, implying that biological processes in which the S. oneidensis Arc system are involved differ from those established in other bacteria drastically (21–24).

**FIG 1:**
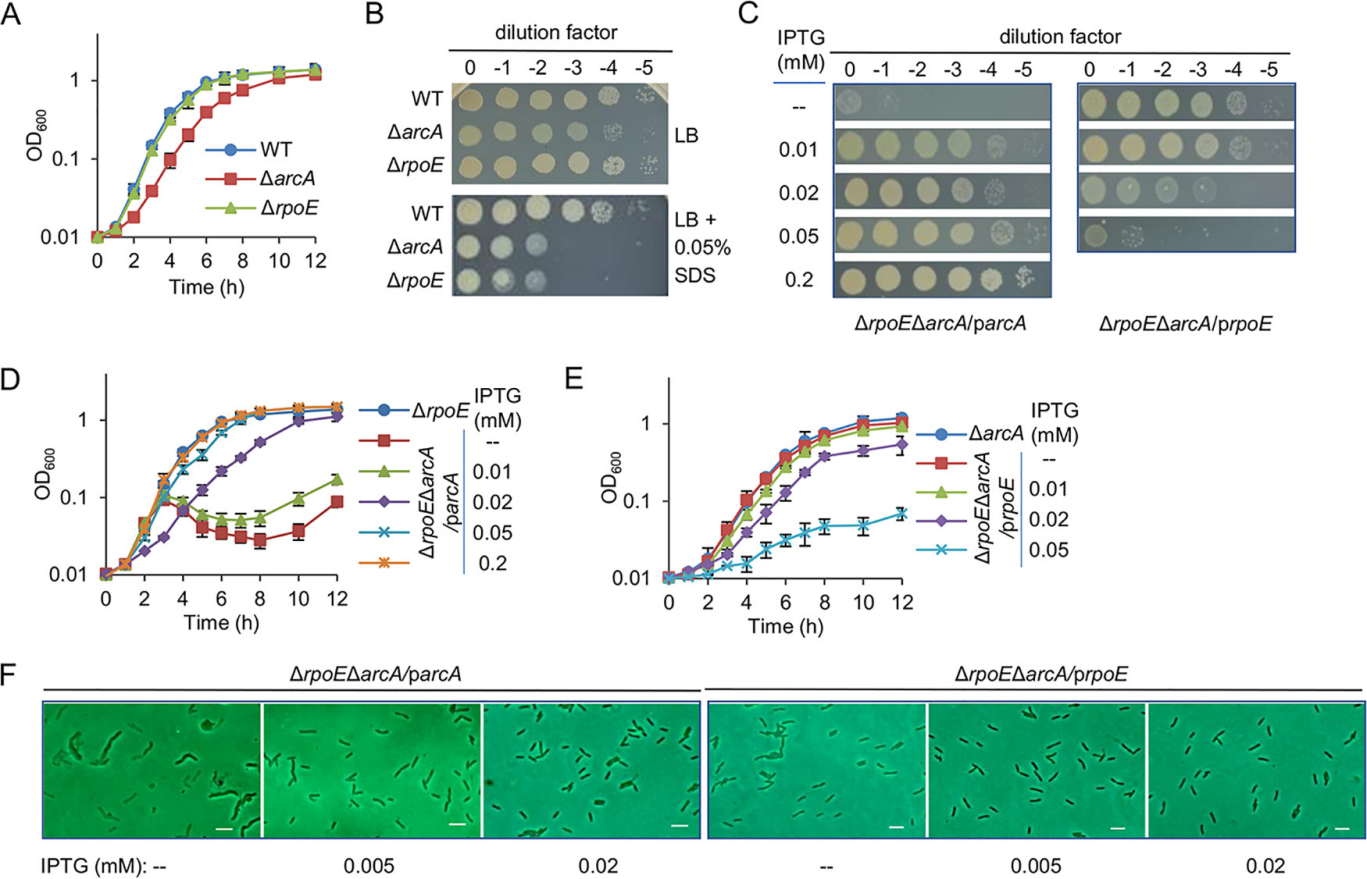
Cell envelope defects likely underpin the synthetic lethal phenotype resulting from the loss of ArcA and σ^E^ in S. oneidensis. (A) Growth of the wild type (WT) and mutants in liquid LB was measured by recording values of OD_600_. Both mutants were verified previously and again by genetic complementation as shown in Fig. S1. (B) Effects of *arcA* or *rpoE* deletion on growth and SDS susceptibility by spotting assays performed on LB agar plates. Cultures of each strain prepared to contain approximately 10^9^ CFU/ml were regarded as the undiluted cultures (dilution factor, 0), which were subjected to 10-fold serial dilution. Five microliters of each dilution was dropped on indicated agar plates and photographed 30 h later. (C) Effects of *arcA* and *rpoE* double deletion on growth. Expression of both genes was driven by IPTG-inducible promoter P*tac*, with IPTG at indicated concentrations. (D) Growth of Δ*rpoE*Δ*arcA*/p*arcA* strain in liquid LB with IPTG at indicated concentrations. The Δ*rpoE*Δ*arcA*/p*arcA* strain was grown overnight in LB liquid containing 0.2 mM IPTG, and the cells were collected by centrifugation, washed twice with fresh IPTG-free LB liquid, and transferred to LB liquid containing IPTG at indicated concentrations to an OD_600_ of 0.01. (E) Growth of Δ*rpoE*Δ*arcA*/p*rpoE* strain in liquid LB with IPTG at indicated concentrations. (F) Cell morphology of relevant strains in LB with IPTG at indicated concentrations by phase-contrast microscope. Cells of the exponential phase (∼0.4 of OD_600_) were fixed on a slice of LB agar. Scale bars, 2 μm. In all panels, experiments were performed at least three times, with either representative data or the means of replicate values ± standard deviations being presented.

In S. oneidensis, Arc was initially identified as a regulator involved in anaerobic respiration because it is required for transcription of the operon for the DMSO reductase and therefore is essential to DMSO respiration (18). Later on, the regulatory role of ArcA was extended to growth supported by aerobic respiration, although the underpinning mechanisms remain incompletely understood (13, 21) (Fig. 1A). Surprisingly, both transcriptomics and proteomics studies have revealed that the genes encoding a large number of membrane proteins, such as FadL (short-chain fatty acid transporter), PspA (phage shock protein), SO_2427 (TonB-dependent receptor), and CsgE (curli assembly/transport protein), to name a few, are among the most differentially expressed upon ArcA loss (21, 25). The involvement of ArcA in maintaining the cell envelope integrity is ultimately confirmed by the finding that its absence induces increased susceptibility to sodium dodecyl sulfate (SDS) (13, 21, 26) (Fig. 1B). More importantly, with respect to the cell envelope integrity and ESR, ArcA interplays with σ^E^ because σ^E^ and ArcA constitute a synthetic lethal pair in S. oneidensis (14). The goal of this study was to probe mechanisms underpinning synthetic lethality of σ^E^ and ArcA and to establish the link between these two transcriptional regulators.

## RESULTS

### Cell envelope defects likely underpin the synthetic lethal phenotype resulting from the loss of ArcA and σ^E^.

To unravel the mechanisms underlying the synthetic lethality of the *arcA* and *rpoE* double deletion mutant (Δ*rpoE*Δ*arcA*), we first comprehensively assessed physiological impacts of ArcA and σ^E^ produced at various levels on this strain. On LB agar plates without IPTG (isopropyl-β-D-thiogalactopyranoside), Δ*rpoE*Δ*arcA* expressing a copy of *arcA* (Δ*rpoE*Δ*arcA*/p*arcA*) under the control of IPTG-inducible promoter P*tac*, which is slightly leaky (P*tac*, ∼30 Miller units) (27, 28), displayed severely impaired growth, whereas Δ*rpoE*Δ*arcA*/p*rpoE* was normal (Fig. 1C). This is expected because the activity of P*tac* in the absence of the inducer is similar to that of the *rpoE* promoter (P*tac*, ∼30 Miller units; P*rpoE*, ∼20 Miller units), whereas the *arcA* promoter is nearly 10 times stronger in the wild-type (WT) cells growing normally (14, 29). In LB, when IPTG was supplemented no more than 0.01 mM, the optical density (OD, 600 nm) of Δ*rpoE*Δ*arcA*/p*arcA* (the inocula were prepared from the culture grown in LB containing 0.2 mM IPTG by centrifugation, washing, and suspension with fresh medium) increased normally for about 4 h and was followed by a significant reduction (Fig. 1D). This pattern coincides with that observed from the S. oneidensis culture grown in the presence of β-lactam antibiotics, suggesting that a large portion of cells lyse during the OD reduction period (30) (see Fig. S2 in the supplemental material). Expression of *arcA* with 0.02 mM IPTG and above suppressed the lysis, and growth was fully restored with 0.2 mM IPTG compared to that of the Δ*rpoE* strain (Fig. 1C and D). In the case of σ^E^, the best growth was observed from *rpoE* expression without IPTG (Fig. 1C and E). σ^E^ production induced by as low as 0.02 and 0.05 mM IPTG significantly impaired growth and nearly completely inhibited growth, respectively. Despite this, OD reduction was not observed in the presence of IPTG at all concentrations tested, implying that the overexpression of σ^E^ does not lead to cell lysis (Fig. 1E).

We then visualized the Δ*arcA*Δ*rpoE* strains expressing *arcA* and *rpoE* at various levels using a phase-contrast microscope. The cell morphology of Δ*rpoE*Δ*arcA*/p*arcA* grown without IPTG was clearly altered, with most cells showing a variety of membrane defects, including distorted shape, formation of blebs, and division/separation failure (Fig. 1F). When *arcA* and *rpoE* were expressed at proper levels (Δ*rpoE*Δ*arcA*/p*arcA*, 0.2 mM IPTG; Δ*rpoE*Δ*arcA*/p*rpoE*, no IPTG), Δ*rpoE*Δ*arcA* cells maintained a normal rod-shaped morphology. These observations suggest that the synthetic lethality resulting from the ArcA and σ^E^ double loss is due to cell envelope damages.

### Altered expression of *lptFG* suppresses synthetic lethality of the *arcA* and *rpoE* mutant.

To identify suppressor genes of the synthetic lethal phenotype of Δ*rpoE*Δ*arcA*, we performed transposon mutagenesis on Δ*rpoE*Δ*arcA*/p*arcA* with mariner-based transposon vector pFAC (31). Δ*rpoE*Δ*arcA*/p*arcA* was used as a parental strain because it has markedly different phenotypes with or without IPTG, allowing us to identify additional mutations that switch the phenotypes in the absence of IPTG. pFAC was chosen since it can be not only used for construction of transposon insertion libraries but also applicable for cryptic operon screening because of an embedded promoter in the transposable sequence (31, 32).

**FIG 2:**
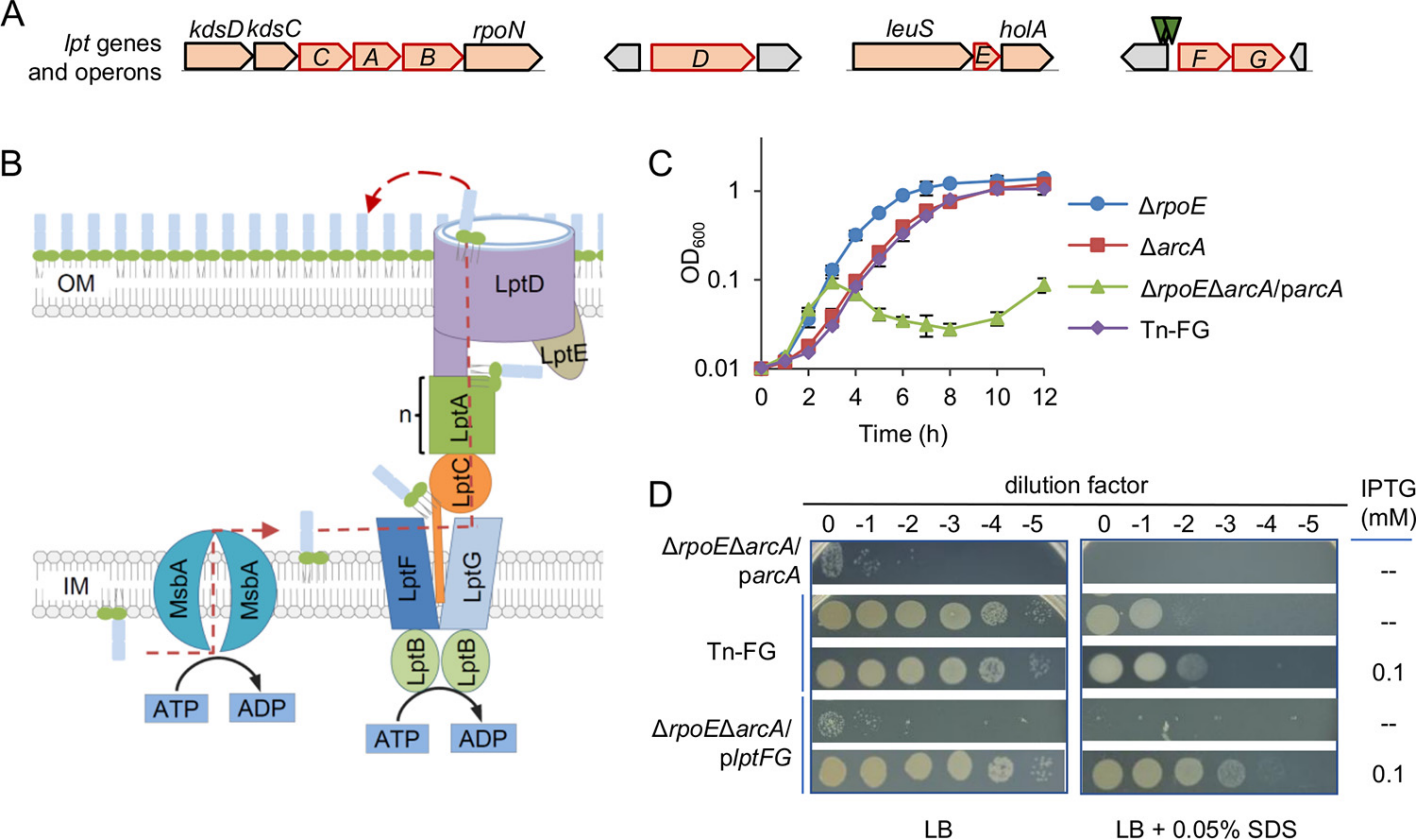
Altered expression of *lptFG* suppresses synthetic lethality of the *arcA* and *rpoE* mutant. (A) Screening for suppressor mutants from Δ*rpoE*Δ*arcA/*p*arcA* in the absence of IPTG was performed by transposon mutagenesis. The transposable element contains a promoter, aiming at expressing genes after the insertion in addition to the gene interruption. Genomic context of the *lptABCDEFG* operons in *S. oneidensis* and insertion sites in Tn-FG suppressor. By genetic mapping, 3 random mutants having insertions in the region upstream of the *lptFG* operon were obtained, which was drawn in scale. Arrows represent the approximate locations of transposon insertions. (B) The working model for LPS transport system based on the E. coli paradigm. (C) The growth of Tn-FG suppressor in IPTG-free LB, which was indistinguishable from Δ*arcA*. (D) Growth and SDS susceptibility of Tn-FG suppressor and Δ*rpoE*Δ*arcA/*p*lptFG* revealed by spotting assays on LB agar plates without or with SDS. In panels C and D, experiments were performed at least three times, with either representative data or the means of replicate values ± standard deviations being presented.

A library of ∼15,000 random mutants were screened for colonies formed on IPTG-free Km^+^Gm^+^ plates that were substantially larger than the average, and more than a hundred were obtained. However, a large majority of these isolates were unstable, losing viability during verification with spotting assays. In the end, only 3 isolates were found to grow well consistently in the absence of IPTG. All of them, which were indistinguishable from one another with respect to growth and SDS sensitivity, had transposon insertions that mapped to the region between *pepA* and *lptF* genes (250 bp upstream of *lptFG* gene), and to simplify description, we named these suppressor strains Tn-FG (Fig. 2A). The *lptF* gene is presumably cotranscribed with *lptG*, both of which encode components of the LPS transport system located on the IM of Gram-negative bacteria (Fig. 2B). LptFG, essential in E. coli, are responsible for the transport of LPS from the IM periplasmic side to LptC in the periplasm (33). Physiological characterization demonstrated that Tn-FG grew indistinguishably from Δ*arcA* but slower than Δ*rpoE* when grown in IPTG-free media (Fig. 2C), indicating that the suppression is on the synthetic lethal phenotype only without correcting the growth defect. This observation is understandable because the defects in growth and cell envelope resulting from the ArcA loss are independent of each other (14). Although Tn-FG was viable with normal cell morphology (see Fig. S4 in the supplemental material), it still carried a cell envelope defect (Fig. 2D), suggesting that the suppression in Tn-FG could not fully correct the envelope defect resulting from the ArcA and σ^E^ double loss.

To validate the suppressing role of LptFG on the synthetic lethal phenotype resulting from the *arcA* and *rpoE* double deletion, we tried to construct Δ*rpoE*Δ*arcA* while expressing *lptFG* from a plasmid and succeeded. The resulting strain, Δ*rpoE*Δ*arcA*/p*lptFG*, was hardly able to grow in IPTG-free media, similar to what was observed for the Δ*rpoE*Δ*arcA*/p*arcA* strain (Fig. 2D). When *lptFG* expression was increased with IPTG induction, Δ*rpoE*Δ*arcA*/p*lptFG* behaved similarly to Tn-FG, with respect to growth and morphology (Fig. 2D; Fig. S3). Notably, the Δ*rpoE*Δ*arcA*/p*lptFG* strain showed increased resistance to SDS, which is likely a result of LptFG overproduction (Fig. 2D). These data indicate that LptFG is crucially involved in the synthetic lethality of the *arcA* and *rpoE* mutation.

### LptFG homeostasis is critical to the integrity of the cell envelope.

**FIG 3:**
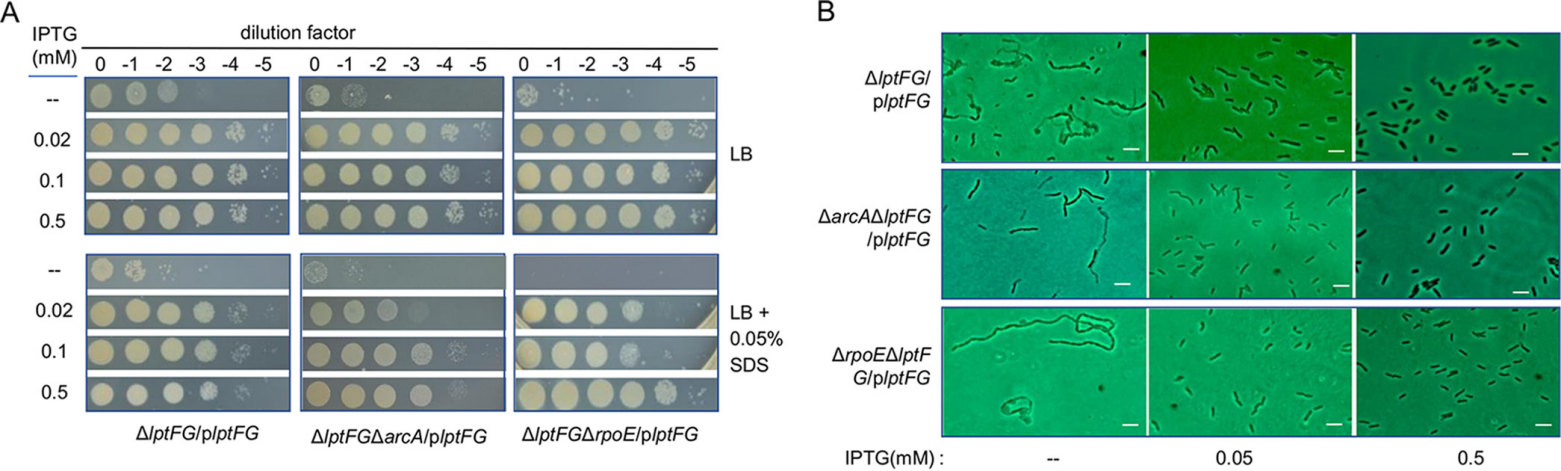
LptFG homeostasis is critical to the integrity of the cell envelope. (A) Growth and viability of relevant strains on LB agar plates without or with SDS. (B) Cell morphology of strains expressing *lptFG* at various levels. Scale bars, 2 μm. In both panels, experiments were performed at least three times, with either representative data or the means of replicate values ± standard deviations being presented.

To explore the effects of LptFG on envelope integrity of the WT, Δ*rpoE*, and Δ*arcA* strains of S. oneidensis, we attempted to delete *lptFG* from them. However, attempts were not successful. Instead, the operon could be removed from strains expressing a copy of the *lptFG* operon under the control of P*tac*. With respect to growth on plates and morphology, the Δ*lptFG*/p*lptFG* strain appeared very similar to Δ*rpoE*Δ*arcA*/p*arcA* (Fig. 3A and B). In the absence of IPTG, cells of Δ*lptFG*/p*lptFG* exhibited severely compromised viability and growth, apparently resulting from substantially impaired cell envelope. When produced sufficiently and even excessively (0.05 mM IPTG and above) (Fig. S4), all of the observed defects were fully corrected (Fig. 3A and B), indicating that the underproduced LptFG (from the leaky promoter, P*tac*, ∼30 Miller units) underlies the phenotypes as well as implying that the overabundance of LptFG alone has no negative effects on the LPS transport in S. oneidensis. When IPTG was not added, Δ*arcA*Δ*lptFG*/p*lptFG* carried defects in viability and growth not significantly different from those of Δ*lptFG*/p*lptFG*, but Δ*rpoE*Δ*lptFG*/p*lptFG* nearly lost viability completely (Fig. 3A). Consistently, Δ*lptFG*/p*lptFG* and Δ*arcA*Δ*lptFG*/p*lptFG* cells had a similar morphology, suggesting that underproduced LptFG may act as a critical factor for the phenotypes of both strains. Furthermore, we found that the cell envelope of Δ*rpoE*Δ*lptFG*/p*lptFG* cells was impaired more severely, evidenced by more blebs and longer cell chains, than that observed in Δ*rpoE*Δ*arcA*/p*arcA* (Fig. 1F; Fig. 3B). These data manifest that σ^E^ is more important than ArcA in protecting cells from cell envelope damages introduced by LptFG in insufficient quantity.

Although the *rpoE* mutant grows indistinguishably from WT under normal conditions, it is more sensitive to SDS (Fig. 1B). To assess whether LptFG homeostasis is critical to the integrity of the cell envelope without σ^E^, we examined the susceptibility of Δ*rpoE*Δ*lptFG*/p*lptFG* to SDS. As shown in Fig. 3A, when LptFG was produced minimally (without IPTG), cells died out on plates with 0.05% SDS. However, in the presence of 0.02 mM IPTG, Δ*rpoE*Δ*lptFG*/p*lptFG* already exhibited growth and viability improved compared to those of Δ*rpoE*. LptFG produced with IPTG at 0.1 mM and above completely corrected the cell envelope defect resulting from the σ^E^ loss (Fig. 1B; Fig. 3A). We also observed that increased LptFG production had no significant effect on SDS susceptibility of the Δ*arcA* strain (Fig. 1B; Fig. 3A). These data indicate that LptFG, crucial to the integrity of the cell envelope in general, is deeply involved in the envelope defect of the *rpoE* mutant in S. oneidensis.

### The shortage of LptFG activates σ^E^ stress response.

**FIG 4:**
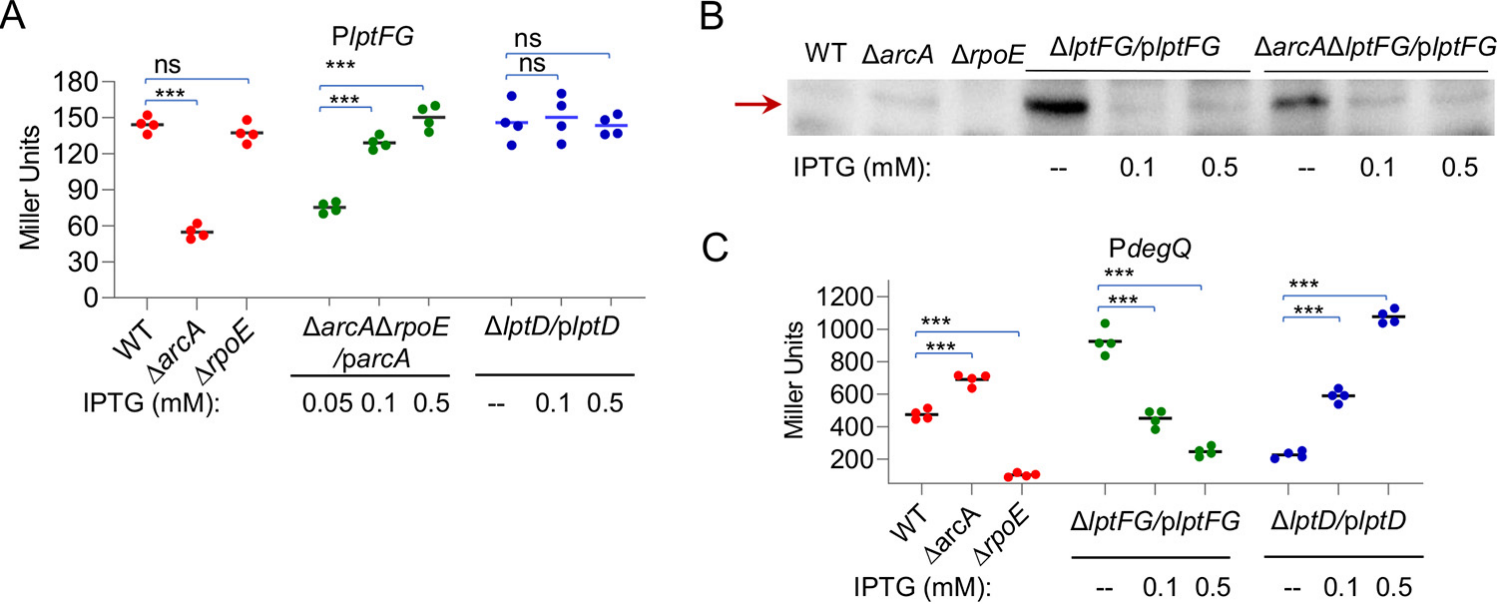
The shortage of LptFG activates σ^E^ stress response. (A) *lptFG* promoter (P*_lptFG_*) activity assay. The activity in cells of the exponential phase (∼0.4 of OD_600_), assayed by integrative *lacZ* reporters, was presented as Miller Units for β-galactosidase activities. In strains expressing relevant genes, expression was controlled with IPTG at indicated concentrations. (B) Western blotting of active σ^E^. Cytoplasmic fractions were prepared from S. oneidensis cells in the exponential growth phase, separated on 15% SDS-PAGE, and electrophoretically transferred to polyvinylidene difluoride (PVDF). σ^E^ was probed with polyclonal antibodies and detected by chemiluminescence. (C) *degQ* promoter (P*_degQ_*) activity assay as carried out in panel A. In panels A and C, asterisks indicate statistically significant difference compared to the wild-type values (ns, not significant; *, *P < *0.05; **, *P < *0.01; ***, *P < *0.001).

Given that the increased LptFG abundance suppresses the synthetic lethal phenotype resulting from the ArcA and σ^E^ loss, we tested whether the depletion of ArcA and σ^E^ influences expression of *lptFG* with an integrative *lacZ* reporter system (34). In line with the prediction by two distinct sources (21, 24) that the *lptFG* operon belongs to the S. oneidensis ArcA regulons, the results of the promoter assay revealed that the activity of the *lptFG* promoter (P*_lptFG_*) was significantly lower in Δ*arcA* than in WT or Δ*rpoE* (Fig. 4A), suggesting that ArcA but not σ^E^ is required for maintaining normal expression of the *lptFG* operon. We also found that the *lptFG* expression in Δ*rpoE*Δ*arcA*/p*arcA* grown without IPTG was similar to that in Δ*arcA*, a result supporting that the loss of *arcA* but not *rpoE* significantly affects *lptFG* expression (Fig. 4A).

We then examined whether the LptFG loss affects expression of *rpoE* given the central role of σ^E^ in maintaining the integrity of the cell envelope. Consistent with the previous findings (14), the *rpoE* promoter activities in Δ*arcA*, Δ*rpoE*, and Δ*rpoE*Δ*arcA*/p*arcA* were very low and rather stable (see Fig. S5 in the supplemental material). Importantly, the loss of LptFG, either alone or with ArcA and σ^E^ together, did not significantly influence the activity of the *rpoE* promoter (Fig. S5). Subsequently, the abundance of active σ^E^ (not membrane associated) was assessed with Western blotting because σ^E^ is activated by accumulation of off-pathway LPS (7). The results showed that the amount of σ^E^ increased in Δ*lptFG*/p*lptFG* cells grown without IPTG relative to that in cells grown with IPTG at 0.1 mM or above (Fig. 4B). Importantly, the quantity of active σ^E^ was further elevated in Δ*arcA*Δ*lptFG*/p*lptFG* cells grown in IPTG-free media. Moreover, we validated the changes in the quantity of the active σ^E^ by using a *lacZ* reporter driven by the *degQ* promoter, which is a verified member of the σ^E^ regulon (14). As expected, the *degQ* promoter exhibited σ^E^-dependent activity and the ArcA loss displayed an upregulating effect (Fig. 4C). When LptFG was present at minimal levels, the highest activity was observed, and the activity decreased with the LptFG amounts inversely (Fig. 4C). These data, collectively, suggest that LptFG at reduced levels triggers the σ^E^ stress response, which lessens the envelope defects resulting from the LptFG shortage. In line with this, the absence of σ^E^ abolishes this protective mechanism, leading to further aggravated cell envelope damage as seen in Δ*rpoE*Δ*lptFG*/p*lptFG* grown without IPTG.

### LptD in excess impairs the cell envelope.

**FIG 5:**
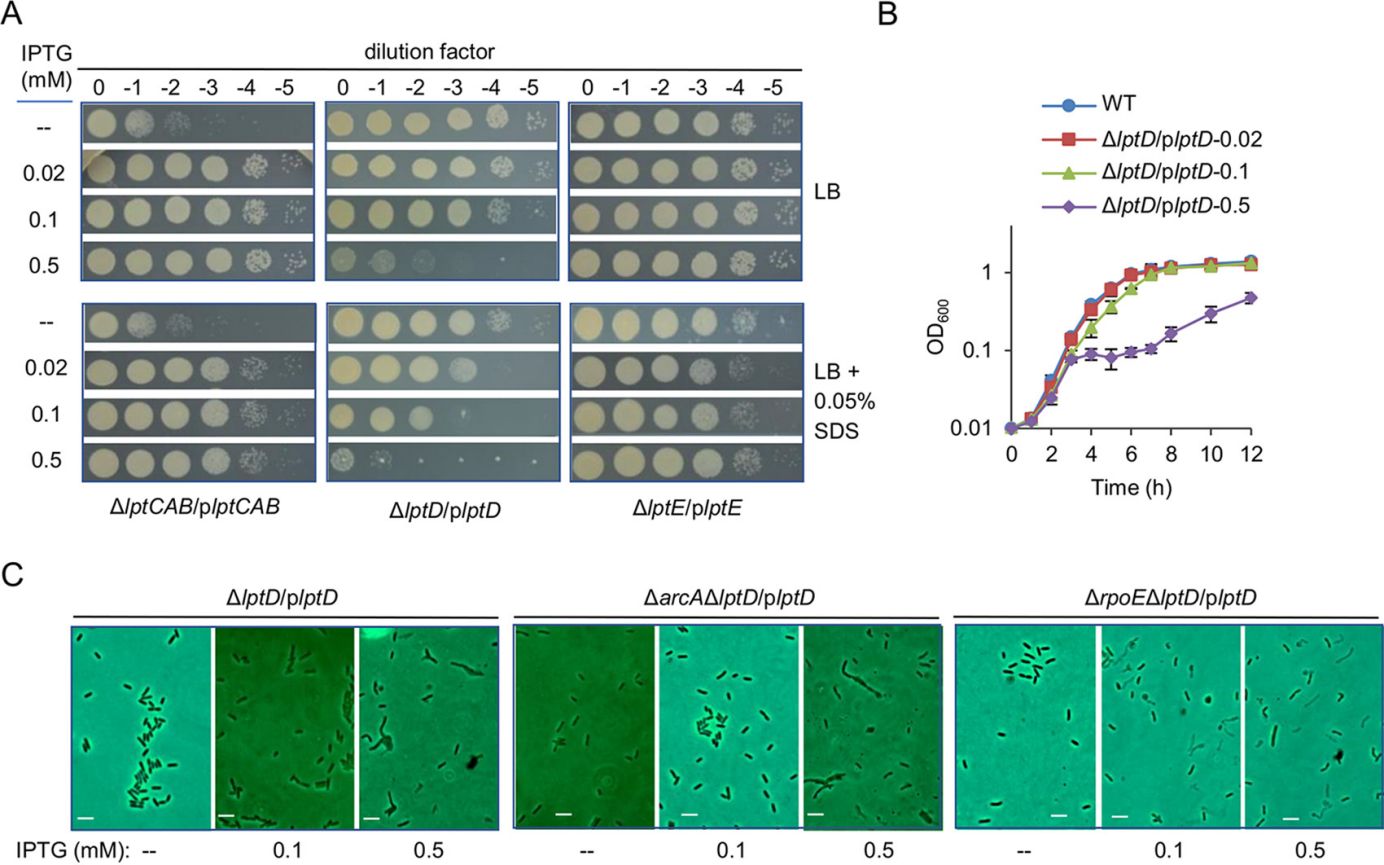
LptD in excess impairs the cell envelope in *S. oneidensis*. (A) Effects of Lpt components in shortage and overexpression on growth, viability, and SDS susceptibility. (B) Growth of strains expressing *lptD* with IPTG at indicated concentrations in LB. (C) Cell morphology of indicated strains expressing *lptD* with IPTG at indicated concentrations. Scale bars, 2 μm. In all panels, experiments were performed at least three times, with either representative data or the means of replicate values ± standard deviations being presented.

The Lpt system is composed of 7 components, including cytoplasmic LptB, LptFG, and LptC in the IM and periplasmic LptA and LptDE in the OM (35–37) (Fig. 2B). To explore whether Lpt components other than LptFG may interplay with ArcA and σ^E^ in S. oneidensis, we tested effects of their absence and overexpression on growth and viability. In strains expressing one of the *lpt* clusters on plasmids, each of them was successfully in-frame deleted, resulting in Δ*lptCAB*/p*lptCAB*, Δ*lptD*/p*lptD*, and Δ*lptE*/p*lptE*. Characterization of these strains revealed that Δ*lptCAB*/p*lptCAB* behaved similarly to Δ*lptFG*/p*lptFG* in terms of growth and viability without and with IPTG (Fig. 5A). However, Δ*lptD*/p*lptD* was normal in the absence of IPTG but showed severe defects in growth and viability with 0.5 mM IPTG, whereas the absence and overproduction of LptE did not exhibit significant influence (Fig. 5A). While results obtained from these strains grown on the plates containing SDS were similar in general, the sensitivity to LptD homeostasis became more evident, noticeable even with IPTG at 0.02 mM (Fig. 5A).

The growth of Δ*lptD*/p*lptD* in liquid media was then assayed. Clearly, when LptD was produced with IPTG at 0.1 mM and above, defects became significant (Fig. 5B). More importantly, LptD in excess by 0.5 mM IPTG induction caused growth curves resembling that of Δ*arcA*Δ*rpoE*/p*arcA*. These defects were likely a result of the substantially impaired cell envelope with excessive LptD (0.5 mM IPTG), evidenced by distorted shape, formation of blebs, dissolving cell membranes, and division failure, coinciding with the morphological changes observed from Δ*arcA*Δ*rpoE*/p*arcA* (Fig. 5C). Altogether, the data suggest that LptD is associated with the phenotypes resulting from the loss of the two regulators.

### Enhanced *lptD* expression partially explains the cell envelope defects in Δ*arcA* and activates σ^E^ stress response.

**FIG 6:**
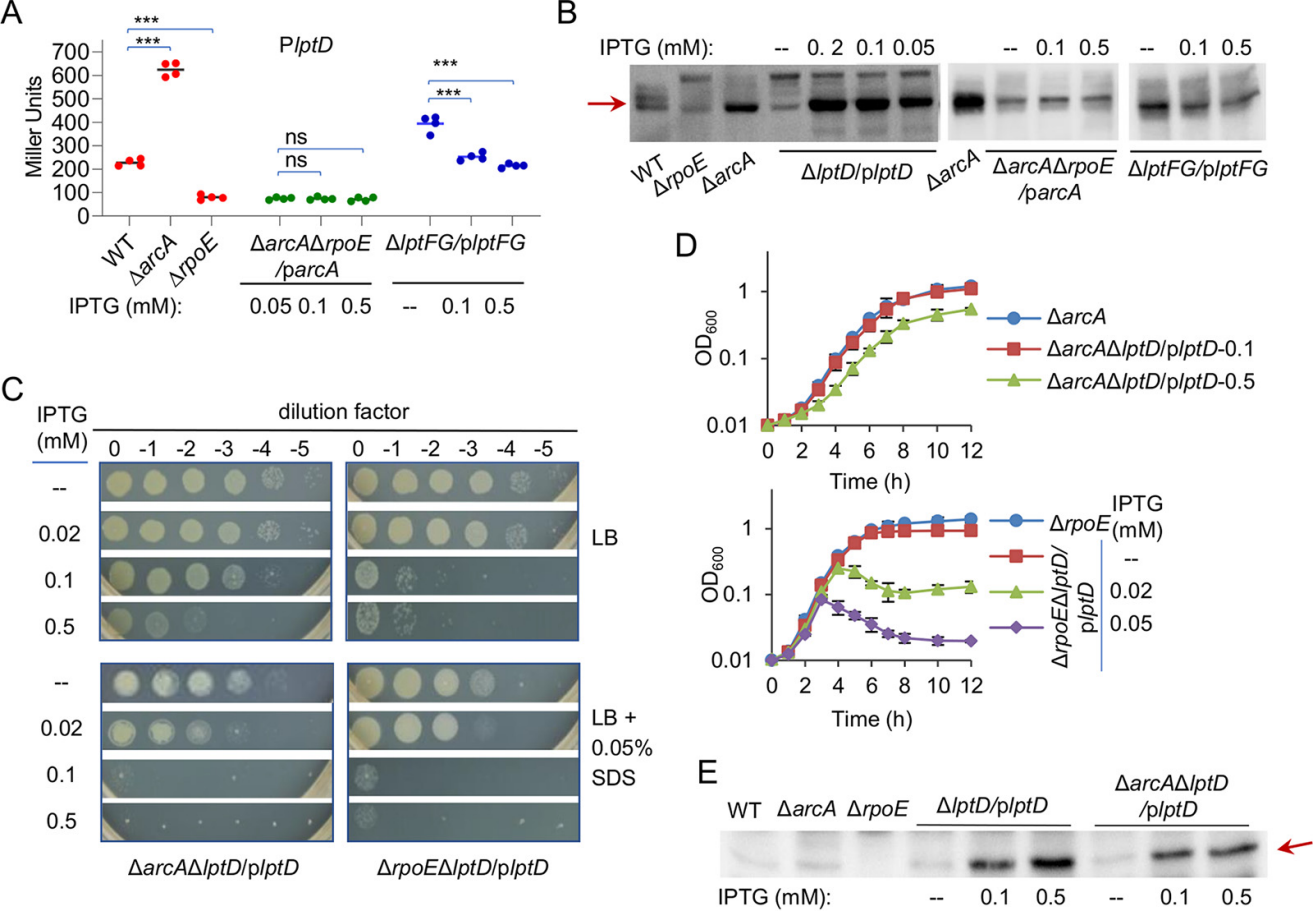
Enhanced *lptD* expression partially explains the cell envelope defects in Δ*arcA* and activates σ^E^ stress response. (A) Effects of ArcA, σ^E^, and LptFG on *lptD* expression by *lacZ* reporter assays. (B) Effects of ArcA, σ^E^, and LptFG on LptD levels by Western blotting. (C) Effects of LptD at various levels on growth, viability, and SDS susceptibility revealed by spotting assays. (D) Effects of LptD at various levels on growth in LB liquid. (E) Activation of σ^E^ by excessive LptD revealed by Western blotting of cytoplasmic σ^E^. In all panels, experiments were performed at least three times, with either representative data or the means of replicate values ± standard deviations being presented.

According to the regulon prediction, the *lptD* operon, but not other *lpt* operons, is likely controlled by σ^E^ directly (14). Given that LptD homeostasis is critical to the cell envelope, we therefore hypothesized that regulation of *lptD* expression by σ^E^, perhaps ArcA too, may be accountable for the synthetic lethal phenotype. To test this, we assayed activity of the *lptD* promoter (P*_lptD_*) in WT, Δ*arcA*, and Δ*rpoE* strains. As shown in Fig. 6A, the σ^E^ loss compromised P*_lptD_* activity significantly, approximately 30% relative to that of the WT, supporting that the *lptD* operon is a member of the σ^E^ regulon. In contrast, we observed substantially increased activity of P*_lptD_* in Δ*arcA*, up to 3-fold. In the absence of σ^E^, the inducing effect of ArcA at any level under test vanished (Δ*arcA*Δ*rpoE*/p*arcA*) (Fig. 6A), supporting that the expression of *lptD* depends on σ^E^. The LptD levels in the relevant strains were then assayed directly with Western blotting. As shown in Fig. 6B, LptD was present in significantly increased and decreased amounts in Δ*arcA* and Δ*rpoE*, respectively. In the absence of σ^E^, the effects of ArcA on LptD levels became neglectable. Complementation of *arcA* and *rpoE* mutations validated that all these observations were due to the missing genes (see Fig. S6 in the supplemental material).

We then tested whether LptD in increased abundance might play a key role in the cell envelope defects of the *arcA* mutant. To this end, we generated Δ*arcA*Δ*lptD*/p*lptD* and monitored its growth and viability with IPTG at various concentrations. As shown in Fig. 6C, when LptD was overproduced with IPTG at 0.1 mM or above, Δ*arcA*Δ*lptD*/p*lptD* displayed reduced growth and viability, similar to those of Δ*lptD*/p*lptD* (Fig. 5A). When grown with 0.1 mM IPTG in the presence of 0.05% SDS, the difference between Δ*arcA*Δ*lptD*/p*lptD* and Δ*lptD*/p*lptD* became evident: Δ*arcA*Δ*lptD*/p*lptD* lost viability completely, whereas the growth and viability of Δ*lptD*/p*lptD* were affected only modestly (Fig. 5A; Fig. 6C). The effects gained support from the results of growth in liquid media (Fig. 6D) and morphological visualization (Fig. 5C). These data, all together, suggest that the detrimental impact of excessive LptD on the cell envelope is likely, at least in part, accountable for the cell envelope defect of the *arcA* mutant.

The expression assay presented above revealed that LptD is produced at a very low level in the absence of σ^E^, contrasting that in the absence of ArcA. To test how LptD at various levels affects the *rpoE* mutant, Δ*rpoE*Δ*lptD*/p*lptD* was generated and characterized. Different from the Δ*arcA*Δ*lptD*/p*lptD* strain, Δ*rpoE*Δ*lptD*/p*lptD* hardly survived in the presence of 0.1 mM IPTG, regardless of SDS (Fig. 6C). Consistently, in the liquid broth containing IPTG at 0.1 mM and above, Δ*rpoE*Δ*lptD*/p*lptD* showed a short period of growth and then lysed nearly completely (Fig. 6D). Thus, it is clear that σ^E^ is required for alleviating the detrimental effect of excessive LptD. Notably, the growth curve of Δ*rpoE*Δ*arcA*/p*arcA* without IPTG resembled that of Δ*rpoE*Δ*lptD*/p*lptD* with 0.05 mM IPTG, further supporting that the increased LptD levels are a critical factor causing the cell envelope defect of the *arcA* mutant.

Given that LptFG at insufficient levels triggers σ^E^ activation, we hypothesized that this is likely to be the case for LptD when it is overproduced. To test this, the levels of σ^E^ in Δ*lptD*/p*lptD* and Δ*arcA*Δ*lptD*/p*lptD* strains were assessed. The results showed that σ^E^ was more abundant in Δ*lptD*/p*lptD* and Δ*arcA*Δ*lptD*/p*lptD* strains with IPTG at 0.1 mM and above than in WT (Fig. 6E). However, when LptD was minimally produced, it did not introduce a significant difference in the amount of activated σ^E^, a result consistent with the finding that LptD activates the σ^E^ stress response only when it is in overabundance. These observations were confirmed with the P*degQ*-*lacZ* reporter (Fig. 4C). We then moved further to test whether activation of σ^E^ by underproduced LptFG and excessive LptD is intertwined. As shown in Fig. 4A, the expression of *lptFG* was not affected significantly when LptD was produced at various levels. In contrast, the minimal production of LptFG resulted in up to 2-fold induced production of LptD, and this effect vanished when it was produced normally or at increased levels (Fig. 6A and B). These data suggest an explanation for the effect of the LptFG shortage on the cell envelope, that is, the underproduced LptFG leads to overproduced LptD, which in turn triggers σ^E^ activation.

## DISCUSSION

In many proteobacteria, the Arc system is a major transcriptional regulator for respiration in response to environmental cues that alter the redox status of the quinol pool, whereas σ^E^ mediates the cell envelope biogenesis and ESR. Seemingly, these two regulatory systems affect distinct physiological processes, and reports about the interplay between them are rare. However, in S. oneidensis, Arc and σ^E^ are functionally intertwined, amounting to the synthetic lethal phenotype in their simultaneous absence (14). In this study, we have unraveled the mechanisms underlying the phenotype, generating three contributions to the current understanding of the Arc and σ^E^ biology. First, we identified the Lpt system as the dictating factor responsible for the synthetic lethality. Second, we demonstrated that LptFG at underproduced levels *per se*, and its direct consequences, including the increased quantity of activated σ^E^ and subsequent LptD overproduction, largely underlies the cell envelope defect of the *arcA* mutant. Third, both the shortage of LptFG and overabundant LptD elicit ESR, during which σ^E^ is required to activate the damage-controlling system for viability.

By using a random mutagenesis to screen for suppressors, we related LptFG to the ArcA and σ^E^ synthetic lethality. In Gram-negative bacteria, Lpt is composed of seven essential lipopolysaccharide transport proteins (LptABCDEFG) that transport LPS from the IM to the cell surface (37) (Fig. 2B). LptB_2_FG form an atypical ABC transporter at the IM, in stable association with LptC, that extracts LPS from the periplasmic leaflet of the IM (38–41). Once extracted, the amphipathic LPS is transported across the aqueous periplasm through the periplasmic bridge formed by LptC, LptA, and LptD (42, 43). Our data indicate that the Lpt-mediated LPS transport pathway of S. oneidensis is highly conserved and each of the components appears to function the same as its counterpart in E. coli. S. oneidensis cells depleted of LptFG are defective in the transport of LPS to the cell surface, presumably leading to disordered OM structure and OM permeability defects the same as those in E. coli (33). In line with functional association, S. oneidensis LptCAB appears to affect LPS transport in a way similar to that of LptFG because reduced LptCAB creates a defect in LPS transport in the same manner as LptFG in E. coli (43). The complex of LptD and LptE at the OM functions in the final stages of assembling LPS into the outer leaflet of the OM. Moreover, S. oneidensis cells with the minimal expression of *lptD*, Δ*lptD*/p*lptD* without IPTG, do not carry an OM defect, and this scenario can be readily explained by the finding that LptD is characterized as a low-abundance protein in E. coli (44, 45) (Fig. 5A). However, when produced excessively, LptD causes severe OM defects, consistent with the previous findings that the alteration of LptD structure or expression amount could affect not only the transport efficiency of LPS but also the OM permeability and membrane structures (46–49). In contrast, the physiological influence of excessive LptE is negligible because its activity is dependent on LptD (35, 50).

Given that it is increased expression rather than interruption of *lptFG* that recovers viability of the *rpoE arcA* mutant, it is clear that LptFG is underproduced in the absence of both ArcA and σ^E^. Further investigations reveal that ArcA is responsible for the expression difference of LptFG because in both Δ*arcA* and Δ*rpoE*Δ*arcA*, but not Δ*rpoE*, expression of *lptFG* is significantly lower than that in WT (Fig. 4A). This is not surprising, as the *lptFG* operon is a member of the S. oneidensis *arcA* regulon predicted by a combination of transcriptomic, *in vitro* DNA-protein interaction and bioinformatics analyses (21, 24, 29).

Unlike the *lptFG* operon, none of the remaining *lpt* operons is under the direct control of ArcA. Despite this, we found that the *lptD* gene is highly induced in the absence of ArcA and this induction depends on σ^E^ (Fig. 6A and B). This observation coincides with the notion that there is a compensatory mechanism in the Lpt system: if one component is disrupted, leading to impaired function of LPS translocation, other components undergo mutation and/or the altered activity to control/overcome the damage (51–53). For example, increased expression of the membrane-associated ABC protein LptB stabilizes C-terminally truncated LptC mutant proteins, thereby allowing the formation of a sufficient number of stable IM complexes to support growth (52). Certain mutations in the LptF periplasmic domain can compensate for defects in LPS transport conferred by the lack of LptC (51). Moreover, alterations in the LptFG coupling helices with the defective LPS transport can be rescued by changing a residue in LptB that is adjacent to functionally important residues in the groove region (53). Our data, however, suggest a twisted form of “compensation,” that is, when produced insufficiently, LptFG induces LptD production (Fig. 6B), which in turn, unfortunately, causes more profound damages on the cell envelope.

In Gram-negative bacteria, the activation of the σ^E^ stress response system is initiated by the presence of misfolded proteins (especially OM porins) in the periplasm, as well as off-pathway intermediates in LPS transport and assembly (7). The data presented here illustrate a close connection between the σ^E^ stress response and the Lpt system defect in S. oneidensis. Both reduced LptFG and excessive LptD result in OM defects that activate the σ^E^ stress response. Interestingly, among all *lpt* operons, *lptA*, *lptB*, and *lptD* belong to the σ^E^ regulon studied to date (54–56). This apparently holds true for S. oneidensis, as the *lptD* gene is predicted to be controlled by a σ^E^-dependent promoter (14), and upon σ^E^ depletion the expression of *lptD* is no longer responsive to cell envelope stress imposed by SDS. Despite this, it should be noted that the minimal expression of *lptD* is allowed in the absence of σ^E^ because cells are viable without σ^E^ but not without LptD.

Based on the data herein, we propose that the cell envelope defect of the *arcA* mutant is due largely to the combined effects of both underproduced LptFG and overproduced LptD. In the presence of σ^E^, ESR functions and the damages resulting from the ArcA loss can be controlled to some extent, allowing viability albeit being sensitive to SDS. In the *arcA rpoE* double mutant cells, although LptD could not be overproduced, LptFG at reduced levels leads to OM defects, which amount to killing because of the lack of the protection of functioning ESR. Thus, given the hypersusceptibility of S. oneidensis cells to LptD overdose, it is more likely that LptD in excess plays a larger role in the cell envelope defect of the *arcA* mutant whereas LptFG in an insufficient amount is responsible for the synthetic lethality of *arcA* and *rpoE*.

The involvement of ArcA in the regulation of LPS synthesis and modification has been reported in other bacteria. Transcription of genes encoding WzzSE and WzzfepE in Salmonella enterica serovar Enteritidis (S. Enteritidis), which control the long O antigen and the very long O antigen, respectively, is mediated by ArcA in response to oxygen availability (57). Additionally, ArcA of S. Enteritidis also modulates *lpxO* expression, resulting in changes in lipid A hydroxylation (58). In the plant pathogen Dickeya dadantii, ArcA activates transcription of *dltB* and *phoS*, whose products are implicated in modification of LPSs (59). In S. oneidensis, although the shortage of LptFG and excessive LptD are largely responsible for the cell envelope defect of the *arcA* mutant, many other membrane proteins may have a role, too. Omics analyses have revealed that membrane-bound proteins make up a large portion of the most downregulated proteins in the *arcA* mutant (21, 25). Eight and 23 out of the top 10 and 30 most downregulated are proteins outside the cytoplasm (see Table S1 in the supplemental material). Conceivably, these proteins (many are β-barrel outer membrane proteins) *per se* and their biogenesis may greatly affect the cell envelope integrity in S. oneidensis. Thus, it seems that the Arc system of S. oneidensis has shifted to modulate the envelope integrity rather than metabolism, as few metabolic genes of the E. coli Arc regulon are found to be controlled by Arc in S. oneidensis (21). More importantly, although the Arc systems of S. oneidensis and E. coli can respond to changes in the redox status to regulate the activity of ArcA, the former may perceive other signals (19, 60). This is inferred from the finding that the redox-sensing PAS domain within ArcS (CaChe-PAS-PAS-HisKA; E. coli ArcB, PAS-HisKA) is functionally dispensable (60). We envision that ArcS uses the Cache domain located in the periplasm to sense extracellular cues and that other domains may be involved. We are working to determine the precise nature of the inducing signal linked to the role of ArcA in modulation of the envelope integrity.

## MATERIALS AND METHODS

### Bacterial strains, plasmids, and culture conditions.

Bacterial strains and plasmids used in this study are listed in Table 1. Information for primers used for generating PCR products is available upon request. Chemicals were obtained from Sigma-Aldrich Co. unless otherwise noted. E. coli and S. oneidensis strains under aerobic conditions were grown in lysogeny broth (LB; Difco, Detroit, MI) medium at 37 and 30°C for genetic manipulation. When needed, the growth medium was supplemented with chemicals at the following concentrations: 2,6-diaminopimelic acid (DAP), 0.3 mM, ampicillin sodium, 50 μg/ml, kanamycin sulfate, 50 μg/ml, and gentamicin sulfate, 15 μg/ml.

**TABLE 1:** Strains and plasmids used in this study

Strain or plasmid	Description	Reference or source
E. coli		
DH5α	Host for cloning	Lab stock
WM3064	Δ*dapA*, donor strain for conjugation	W. Metcalf, UIUC
S. oneidensis strains		
MR-1	Wild type	Lab stock
HG1342	Δ*rpoE* derived from MR-1	14
HG3988	Δ*arcA* derived from MR-1	21
HG1342-3988c1	Δ*rpoE*Δ*arcA*/p*rpoE* derived from MR-1	14
HG1343-3988c2	Δ*rpoE*Δ*arcA/*p*arcA* derived from MR-1	This study
HG1342-3988c3	Δ*rpoE*Δ*arcA/*p*lptFG* derived from MR-1	This study
Tn-FG	Suppressor derived from Δ*rpoE*Δ*arcA*/p*arcA*	This study
HG1173c	Δ*lptE*/p*lptE* derived from MR-1	This study
HG1369-70c	Δ*lptFG*/p*lptFG* derived from MR-1	This study
HG3636c	Δ*lptD*/p*lptD* derived from MR-1	This study
HG3858-60c	Δ*lptCAB*/p*lptCAB* derived from MR-1	This study
HG1342-1369-70c	Δ*rpoE*Δ*lptFG*/p*lptFG* derived from MR-1	This study
HG3988-1369-70c	Δ*arcA*Δ*lptFG*/p*lptFG* derived from MR-1	This study
HG1342-3636c	Δ*rpoE*Δ*lptD*/p*lptD* derived from MR-1	This study
HG3988-3636c	Δ*arcA*Δ*lptD*/p*lptD* derived from MR-1	This study
Plasmid		
pHGM01	*att*-based suicide vector, Ap^r^, Gm^r^, Cm^r^	61
pHGEI01	Km^r^, integrative *lacZ* reporter vector	34
pHGEN-Ptac	Km^r^, IPTG-inducible expression vector	28
pBBR-Cre	Sp^r^, helper plasmid for antibiotic cassette removal	32
pFAC	Gm^r^, vector containing transposable sequence	31
pHGEI01-P*rpoE*	P*rpoE*-*lacZ* fusion within pHGEI01	14
pHGEI01-P*lptFG*	P*lptFG*-*lacZ* fusion within pHGEI01	This study
pHGEI01-P*lptD*	P*lptD*-*lacZ* fusion within pHGEI01	This study
pHGEI01-P*degQ*	P*degQ*-*lacZ* fusion within pHGEI01	This study
pHGEN-Ptac-*rpoE*	P*tac*-*rpoE* within pHGEN-Ptac	14
pHGEN-Ptac-*lptFG*	P*tac*-*lptFG* within pHGEN-Ptac	This study
pHGEN-Ptac-*lptD*	P*tac*-*lptD* within pHGEN-Ptac	This study
pHGEN-Ptac-*lptE*	P*tac*-*lptE* within pHGEN-Ptac	This study
pHGEN-Ptac-*lptCAB*	P*tac*-*lptCAB* within pHGEN-Ptac	This study

### Mutant construction and complementation.

In-frame deletion strains for S. oneidensis were constructed using the *att*-based fusion PCR method as described previously (61). In brief, two fragments flanking the gene of interest were amplified independently and then joined together by a second round of PCR. The resulting fusion fragment was introduced into suicide plasmid pHGM01 by site-specific recombination using the BP Clonase (Invitrogen), and the resulting mutagenesis vectors were maintained in *E. coli* DAP-auxotroph WM3064. The vectors were then transferred from *E. coli* into the relevant *S. oneidensis* strain by conjugation. Integration of the mutagenesis construct into the chromosome was selected by gentamicin resistance and confirmed by PCR. For most of mutants constructed in this study, plasmid pHGEN-Ptac expressing a copy of the target gene under the control of isopropyl β-d-1-thiogalactoside (IPTG)-inducible promoter P*tac* was introduced to verified transconjugants before resolution (28). Such transconjugants were grown in LB with IPTG at proper concentrations in the absence of NaCl and plated on LB supplemented with 10% sucrose for resolution. Gentamycin-sensitive and sucrose-resistant colonies were screened by PCR for deletion of the target gene. Mutants were verified by sequencing the mutated regions.

For genetic complementation of the mutants and inducible gene expression, genes of interest generated by PCR were cloned into pHGEN-Ptac (28). After verification by sequencing, the resultant vectors in E. coli WM3064 were transferred into the relevant strains via conjugation.

### Spotting assays.

Spotting assays were employed to evaluate viability, growth inhibition, and SDS susceptibility of relevant S. oneidensis strains on LB plates. Cells of the exponential phase (∼0.4 of OD_600_, the same throughout the study unless otherwise noted) were collected by centrifugation and adjusted to 10^8^ CFU/ml, which was set as the undiluted culture (dilution factor 0). Ten-fold serial dilutions were prepared with fresh medium. Five microliters of each dilution was dropped onto LB plates without or with SDS. The plates were incubated for 30 h before being read. All experiments were conducted at least three times.

### Transposon mutagenesis and screening.

A random mutation library for the Δ*rpoE*Δ*arcA*/p*arcA* strain was constructed with pFAC, which is a transposon vector with a promoter embedded in the transposable region (31, 32). Synthetic lethality of the *arcA* and *rpoE* suppressor strains were selected from a library of ∼15,000 random mutants for colonies formed on IPTG-free plates containing kanamycin and gentamicin that were substantially larger than the average. Spotting assays were then performed to verify these transposon mutants. Those that consistently grew well in the absence of IPTG were subjected to the mapping of the transposon insertion sites by using the arbitrary PCR (62).

### Analysis of gene expression.

Activity of target promoters was assessed using a single-copy integrative *lacZ* reporter system as described previously (63). Briefly, fragments containing the sequence (∼300 bp) upstream of the target operon were amplified, cloned into the reporter vector pHGEI01, and verified by sequencing. The resultant vector in E. coli WM3064 was then transferred by conjugation into relevant S. oneidensis strains, in which it integrated into the chromosome, and the antibiotic marker was removed subsequently (32). Cells of the exponential phase under test conditions were harvested by centrifugation, washed with phosphate-buffered saline (PBS, pH 7.0), and lysed with the lysis buffer (0.25 M Tris/HCl [pH 7.5], 0.5% Triton X-100). We collected the resulting soluble protein after centrifugation and used it for enzyme assay by adding the aliquot of the *o*-nitrophenyl-β-d-galactopyranoside (ONPG) (4 mg/ml). β-Galactosidase activity was determined by monitoring color development at 420 nm using a Synergy 2 Pro200 multi-detection microplate reader (Tecan), and results were presented as Miller units.

### Microscopic analysis.

Motic BA410E phase-contrast microscope was used to visualize the morphological changes of S. oneidensis cells. Cells of the exponential phase were fixed on a slice of LB agar and visualized in a time course manner. Micrographs were captured with a Moticam ProS5 Lite camera and Motic images plus 3.0 software.

### Western blotting.

Rabbit polyclonal antibodies against S. oneidensis LptD, which were prepared using a synthesized fragment (amino acids [aa] 40 to 198) as the antigen in accordance with standard protocols provided by the manufacturer (GenScript, Nanjing, China), and antibodies against σ^E^ prepared previously (14) were used for immunoblotting analysis. Sample preparations, including cell cultivation and subcellular fractionation, were carried out as described before (14, 63). Throughout this study, the total protein concentration of the cell lysates was determined by the bicinchoninic acid assay (Pierce Chemical). The resulting samples for defection of LptD and σ^E^ were subjected to electrophoresis on 6% and 15% SDS polyacrylamide gels (PAGE), respectively. Proteins were transferred to polyvinylidene difluoride (PVDF) membranes for 1 h at 60 V using a Criterion blotter (Bio-Rad). The blotting membrane was probed with specific antibodies, followed by a 1:10,000 dilution of goat anti-rabbit immunoglobulin G-alkaline phosphatase conjugate. The alkaline phosphatase was detected using a chemiluminescence Western blotting kit (Roche Diagnostics) in accordance with the manufacturer’s instructions. Images were visualized with Clinx Imaging System (Clinx, Shanghai, China).

### Other analyses.

Experimental values were subjected to statistical analyses and presented as means ± standard error of the mean (SEM). Student’s *t* test was performed for pairwise comparisons of groups.
