# Supplementary material for: Lipopolysaccharide Transport System Links Physiological Roles of σE and ArcA in the Cell Envelope Biogenesis in Shewanella oneidensis
Source: Microbiol Spectr. 2021 Aug 18;9(1):10.1128/spectrum.00690-21. doi: 10.1128/spectrum.00690-21 (PMC8552667; doi:10.1128/spectrum.00690-21)
Supplement: SUPPLEMENTAL FILE 1 — Supplemental material. Download SPECTRUM00690-21_Supp_1_seq1.pdf, PDF file, 0.3 MB [file spectrum00690-21_supp_1_seq1.pdf]

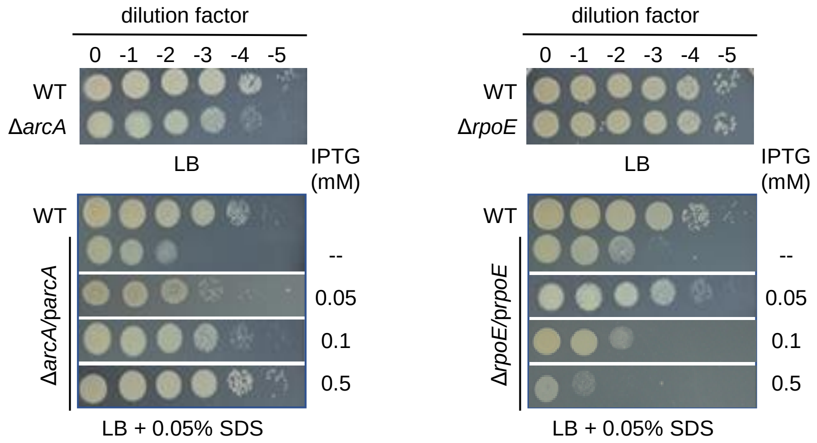

FIG S1. Effects of ArcA and RpoE produced at varying levels on the SDS susceptibility of Δ*arcA* and Δ*rpoE* respectively. Expression of both genes was driven by IPTG-inducible promoter Ptac, with IPTG at indicated concentrations.

A

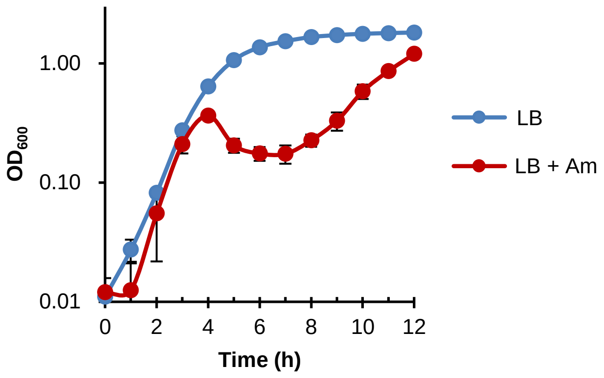

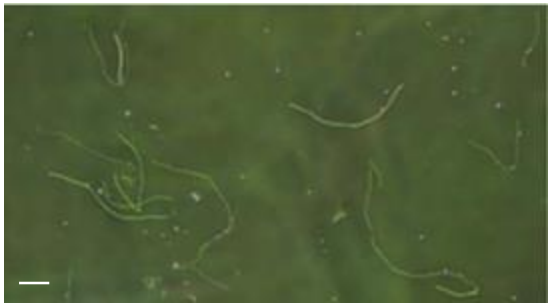

FIG S2. Growth and morphology of *S. oneidensis* in the presence of ampicillin. Fresh LB containing 2.5 μg/ml ampicillin was inoculated to approximately 0.01 of OD_{600} by overnight cultures and incubated at 30 ^{o}C in a shaker at 200 rpm. Growth (left panel) was monitored and morphology (right panel) was examined. Scale bar, 2 μm.

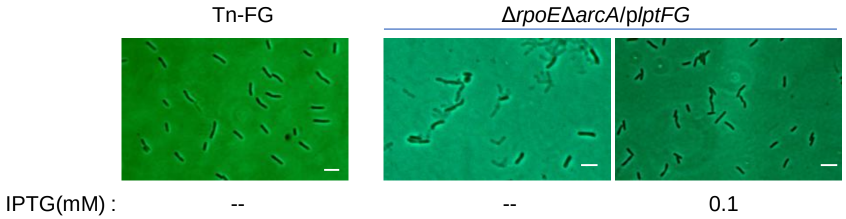

FIG S3. Morphology of Tn-FG and Δ*rpoE*Δ*arcA*/p*lptFG.* Scale bar, 2 μm.

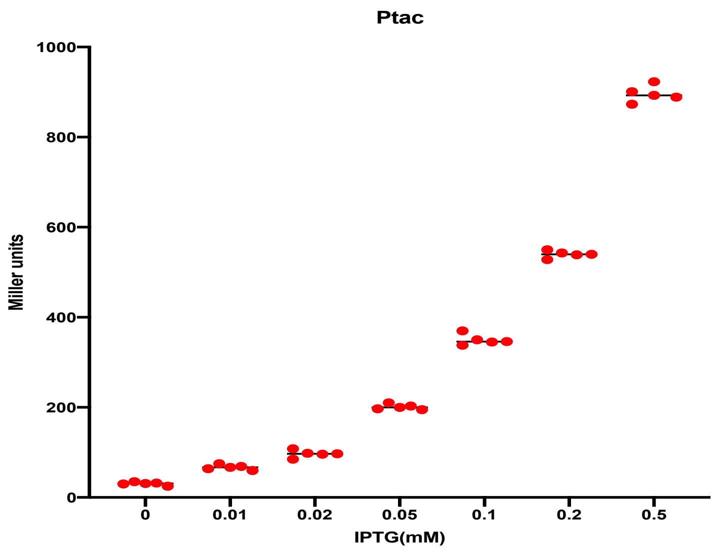

FIG S4. Calibration of the vector with the *E. coli lacZ* gene.
The full-length *lacZ* was placed at MCS and the resulting plasmid was introduced to the *S. oneidensis* wild-type strain. The mid-log phase cultures were induced by IPTG at indicated concentrations for 2 h, and then cells were collected for β-galactosidase activity assay. Experiments were performed at five times, with all data presented.

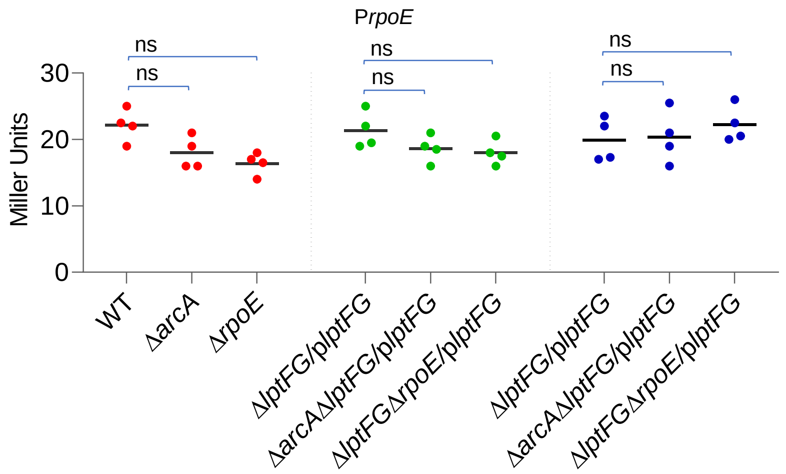

Fig S5. Expression of *rpoE*. Samples were prepared with cells of indicated strains grown to the exponential phase with 0.05 mM IPTG. Samples were divided into two parts: one was immediately applied to the P*rpoE* activity (P*rpoE*) assay and the other was subjected to removal of IPTG first and the P*rpoE* activity assay was performed 2 hours later (before the cells start to lyse). The promoter activity of *rpoE* was assayed by integrative *lacZ* reporter. Asterisks indicate statistically significant difference compared to the wild-type values (ns, not significant; *, $P < 0.05$; **, $P < 0.01$; ***, $P < 0.001$).

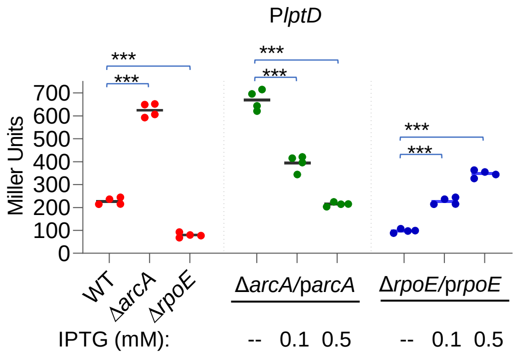

Fig S6. Expression of *lptD*. Samples were prepared with cells of indicated strains grown to the exponential phase. The promoter activity of *rpoE* (P*rpoE*) was assayed by integrative *lacZ* reporter. Asterisks indicate statistically significant difference compared to the wild-type values (*, $P < 0.05$; **, $P < 0.01$; ***, $P < 0.001$).
